# Ink-Based Additive Manufacturing of a Polymer/Coal Composite: A Non-Traditional Reinforcement

**DOI:** 10.1021/acsaenm.4c00126

**Published:** 2024-05-06

**Authors:** Barath Sundaravadivelan, Dharneedar Ravichandran, Anna Dmochowska, Dhanush Patil, Sri Vaishnavi Thummalapalli, Arunachalam Ramanathan, Jorge Peixinho, Guillaume Miquelard-Garnier, Kenan Song

**Affiliations:** † Department of Mechanical Engineering, School for Engineering of Matter, Transport, and Energy, Ira A. Fulton Schools of Engineering, Arizona State University, Tempe, Arizona 85281, United States; ‡ School of Manufacturing Systems and Networks Ira A. Fulton Schools of Engineering, Arizona State University, Mesa, Arizona 85212, United States; § Laboratoire PIMM, CNRS, Arts et Métiers Institute of Technology, Cnam, HESAM Universite, 75013 Paris, France; ∥ School of Environmental, Civil, Agricultural, and Mechanical Engineering (ECAM), College of Engineering, University of Georgia, Athens, Georgia 30602, United States

**Keywords:** sustainability, coal reinforcement, 3D printing, composites, mechanical properties

## Abstract

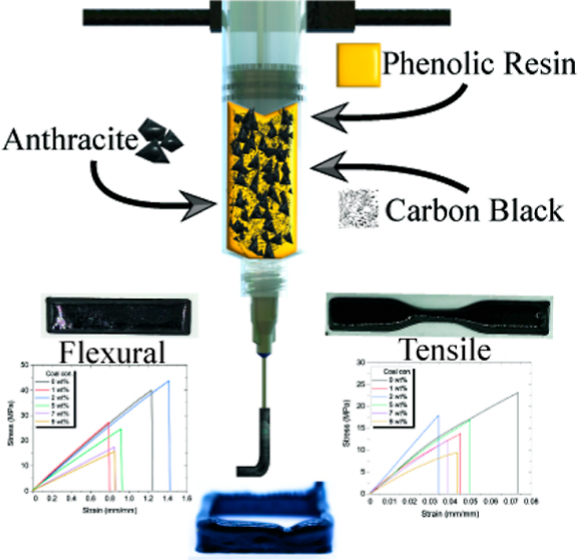

Coal, a crucial natural resource traditionally employed for generating carbon-rich materials and powering global industries, has faced escalating scrutiny due to its adverse environmental impacts outweighing its utility in the contemporary world. In response to the worldwide shift toward sustainability, the United States alone has witnessed an approximate 50% reduction in coal consumption. Nevertheless, the ample availability of coal has spurred interest in identifying alternative sustainable applications. This research delves into the feasibility of utilizing coal as a nonconventional carbon-rich reinforcement in direct ink writing (DIW)-based 3D printing techniques. Our investigation here involves a thermosetting resin serving as a matrix, incorporating pulverized coal (250 μm in size) and carbon black as the reinforcement and a viscosity modifier, respectively. The ink formulation is meticulously designed to exhibit shear-thinning behavior essential for DIW 3D printing, ensuring uniform and continuous printing. Mechanical properties are assessed through the 3D printing of ASTM standard specimens to validate the reinforcing impact. Remarkably, the study reveals that a 2 wt % coal concentration in the ink leads to a substantial improvement in both tensile and flexural properties, resulting in enhancements of 35 and 12.5%, respectively. Additionally, the research demonstrates the printability of various geometries with coal as reinforcement, opening up new possibilities for coal utilization while pursuing more sustainable manufacturing and applications.

## 1 Introduction

Over the past decade, there has been a consistent downward trend in coal usage for household and industrial energy applications, driven by efforts to combat global warming attributed to CO_2_ emissions and other greenhouse gases. In the United States alone, there has been a substantial 50% reduction in the use of coal for energy production.^1^ The rise of renewable energy technologies has further contributed to making cleaner and more cost-effective alternatives, such as solar and wind power, increasingly attractive in comparison to coal and natural gas power plants.^2^ Despite the environmental concerns associated with coal mining, it remains a complex issue intertwined with economic and political considerations, employing over 4.5 million people globally.^3^ Moreover, coal mining yields valuable byproducts like natural gas, pyrite, sulfur, and trace metals, including rare earth elements extracted during coal processing.^4,5^ Coal finds essential applications beyond energy production, playing a pivotal role in steel manufacturing through the production of coke,^6^ cement,^7^ ammonia sourcing,^8^ and carbon-based products like activated carbon^9^ and carbon black (CB).^10^ Additionally, coal tar pitch derived from coal is a crucial component in asphalt.^11^ Notwithstanding these varied applications, coal remains a primary energy source in developing countries due to its widespread availability.^12^ While a complete cessation of coal production and utilization may prove impractical, there is a growing emphasis on reducing its use, especially in nonessential contexts. Aligned with the 2030 Agenda for Sustainable Development, the United Nations has established 17 Sustainable Development Goals. Among these, three goals, either directly or indirectly, aim to achieve a minimum of 55% reduction in greenhouse gas emissions from 1990 levels.^13^ Meeting this target necessitates will have a significant reduction in reliance on carbon-intensive energy sources, such as coal.

An alternative application of coal and its byproducts lies in their utilization as reinforcements in the production of composite materials. While the incorporation of coal as an additive or reinforcing material is not a novel concept, its use in polymer composites has garnered attention. Carbon fibers, derived from coal-based precursors like coal tar pitch^14^ or polyacrylonitrile,^15,16^ derived through suitable solvent extraction and proper heat treatment processes, are commonly employed as reinforcements in these composites. Additionally, carbon nanotubes can be synthesized through potassium-catalyzed pyrolysis of coal.^17^ Various carbon-based particles such as graphene, CB, and fly ash can also be derived from coal, serving as source materials for reinforcing composites.^18^ These materials are strategically introduced into the polymer matrix to enhance the mechanical strength, electrical properties, thermal conductivity, and other functional aspects of the composite.^19^ While traditional manufacturing processes for high-performance carbons are known for being time-consuming and expensive, coal can offer a more direct and cost-effective approach.^20−22^ In contrast to the intricate procedures involved in producing carbon materials, coal can be directly employed as a filler material, enhancing mechanical properties, UV, and thermal stability. Notably, coal exhibits a high filler loading capacity, allowing for the customization of composites based on specific application requirements. Moreover, the pre- and postprocessing costs associated with coal are comparatively lower than those of other nanoparticle processing methods, making it an appealing raw material for composite production.^23−25^

Additive manufacturing emerges as a technology that can effectively utilize coal as a reinforcing material in the production of carbon-reinforced polymer composites, owing to its versatile array of mechanisms.^26^ Often referred to as 3D printing, additive manufacturing is a cutting-edge technique that constructs three-dimensional structures layer by layer, providing unparalleled design freedom and complexity. This technology has found applications across diverse industries, including aerospace, automotive, healthcare, and consumer goods.^27−29^ The landscape of manufacturing is transforming with continuous enhancements in materials, techniques, and technology, leading to reduced costs, time efficiency, and the promotion of sustainable manufacturing practices. Among the various additive manufacturing mechanisms, direct ink writing (DIW) stands out as a versatile approach. DIW involves depositing viscoelastic liquids such as polymer or particle-suspended solutions or gels in a continuous flow to create 3D structures. Notably, DIW offers several advantages over other additive manufacturing techniques, with versatility being a key highlight.^30^ Through the meticulous formulation of a shear-thinning feedstock with a polymer-based matrix, DIW allows the incorporation of nanoparticles of different dimensions (e.g., 1D or 2D) at varying concentrations. Moreover, it provides flexibility in adopting different curing strategies, including thermal or UV-based methods.^31^ DIW boasts shorter feedstock formulation and production times compared to other 3D printing mechanisms, making it a highly suitable technology for on-the-fly manufacturing. DIW excels in constructing structures with intricate geometries, showcasing proficiency in creating overhangs and internal voids. This capability makes it particularly suitable for generating specialized structures such as lattices, interwoven designs, or meta-structures, especially in biomedical applications.^32−35^

This study investigates the potential of coal as an economical and sustainable substitute for traditional carbon reinforcements in ink-based additive manufacturing. The primary matrix comprises a thermosetting polymer-based resin, specifically phenol-formaldehyde (PF), while high carbon content anthracite coal (≪90% coal concentration) serves as the reinforcement and CB functions as a viscosity modifier. Employing the DIW 3D printing mechanism, the manufacturing process involves instantaneous thermal curing of the inks to create 3D structures. Through precise formulation, inks with varying concentrations of dispersed coal (from 1 to 9 wt %) were developed and assessed for changes in material properties, including Young’s modulus, ultimate tensile strength (UTS), and bending strength. In-depth curing studies are conducted to optimize the resin’s pot life, achieve maximum property enhancement, and enable instantaneous part production. The study also highlights the possibility of printing free-standing structures for different geometries and thin wall structures, highlighting the shape fidelity of coal composite inks for 3D printing and potential future applications such as localized space manufacturing.

## 2 Results and Discussion

### 2.1 Curing Kinetics

**Figure 1:**
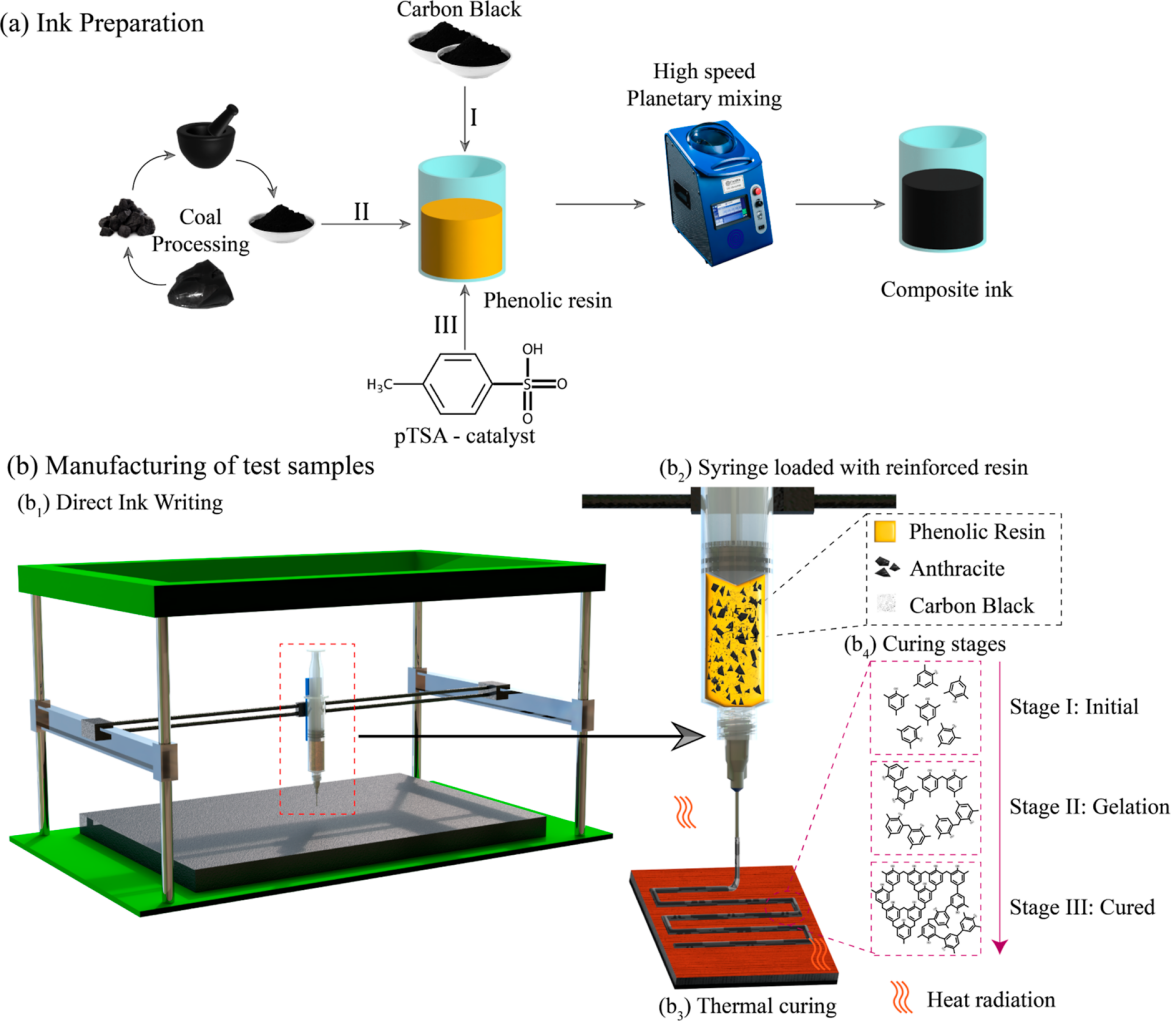
(a) Ink preparation steps: (I) adding a viscosity modifier, specifically CB, (II) preprocessing coal into fine particles and incorporating them into the resin/CB mixture to create composite ink, and (III) introducing a catalyst to accelerate the curing process. (b) Manufacturing procedure overview: (b1) DIW 3D printing platform, (b2) DIW syringe loaded with particle-reinforced phenolic resin, (b3) heated build plate utilized for curing, and (b4) sequential stages of curing required to form a rigid structure.

The resole-type phenolic resin used in this study cures through condensation polymerization, during which the resin monomers, namely, phenol and formaldehyde, form a 3D network structure. The curing is initiated by introducing heat, usually a slow cure process. During the curing process, the resin undergoes further condensation reactions, which involve the elimination of water molecules and the formation of methylene and methylene ether bridges between phenol units. This curing can be accelerated by adding an alkaline or acidic source based on the chemistry of the resin.^36^ In this study, an organic acid (pTSA) was added to the J2027L phenolic resin (Figure 1a). The H^+^ ions in the acid promote the nucleophilic formation of ionic phenol and formaldehyde compounds, which leads to the formation of methylene bridges (−CH_2_−), through which the phenol and formaldehyde molecules form a 3D intricate cross-linking (Figure 1b).^37,38^

The initial curing studies were conducted with varying concentrations of pTSA on a hot plate. The temperature and time to cure are noted. From the observation, the resin with no catalyst takes about 7 min to cure at 150 °C. Meanwhile, the resin with 1, 2, and 3 wt % pTSA cures within ∼6, 3 min, and ≪30 s at 150, 100, and 90 °C, respectively. The experimental results of the curing temperature were confirmed by differential scanning calorimetry (DSC) analysis (Figure S1), which helped identify the shift in the curing peak for different concentrations of pTSA.

As seen in Figure S1, at 0 and 1 wt % pTSA concentrations, no significant change in curing temperatures was noticed during this heating cycle. Additionally, a step in the heat flow signal was observed above 100 °C, most likely correlated with water evaporation in the resin. At even higher temperatures, the artifacts attributed to changes in the crucible pressure were noted. Meanwhile, with 2% and 3% pTSA, the curing temperatures match the initial observations from the hot plate experiments. The resin with 2 wt % pTSA cures at around 94 °C and with 3% pTSA at approximately 82 °C. Even though the print bed temperature can reach up to 120 °C the curing of the resin with 3 wt % pTSA can happen too quickly, reducing the pot life of the resin significantly due to the initiation of the curing chain reaction when exposed to heat. Hence, 2 wt % pTSA with a print bed temperature of 95 °C was considered suitable.

### 2.2 Ink Formulation and Manufacturing

In ink-based additive manufacturing, it is important to understand the material’s flow behavior, which also determines printability. For DIW 3D printing, it is essential that the ink exhibits a shear thinning behavior; meaning, its viscosity decreases with increasing shear rate, allowing for smooth flow of the ink through the nozzle. To investigate the flow behavior of materials under the effect of an applied force, rheological tests were conducted. The inks with different concentrations of reinforcements were studied to observe their impact on the rheological response.

The phenolic resin, when only mixed with the catalyst, exhibited low viscosity, resulting in inconsistent printing structures (Figure S2). Specifically, a viscosity equal to 1 Pa·s was insufficient for producing a uniform and continuous ink flow necessary for well-integrated structures, aligning with findings from other studies.^39,40^ To increase the viscosity, CB was used as a viscosity modifier. Figure S2 illustrates that a concentration of 12 wt % CB in the resin increases the viscosity to 173 Pa·s at a shear rate of 0.1 s^–1^ and 29 Pa·s at 1 s^–1^, achieving the necessary consistency for continuous and stable ink flow. Additionally, the inclusion of CB aids in the dispersion and suspension of coal in later stages. Consequently, the coal composite inks were prepared with a constant 2 wt % concentration of the acid catalyst (pTSA) and 12 wt % CB as a viscosity modifier. Samples labeled as 0 wt % coal are PF with 12 wt % CB; hence, the results reflect the effect of CB as a reinforcement. While CB is used as a viscosity modifier in this study, the incorporation of CB can also lead to improvements in mechanical, thermal, or electrical properties.^41^ It is a popular choice of reinforcement in the rubber manufacturing industry to modify the properties of elastomer-based composites.^42^

Various concentrations of coal were added to the ink. Note that the coal used in this study has a purity of 97.36%, as confirmed by thermogravimetric analysis (TGA), meaning it has fewer impurities and the properties are influenced based on this, as depicted in Figure S3. As seen in Figure 1a, the obtained coal was first pulverized into small particles with an average size of 250 μm through hammering and later using a mortar and pestle. A sieve with a 250 μm size was used to separate the bigger particles, and later, the uniform coal particles were added to the resin along with other components, i.e., CB and catalyst, and mixed uniformly one after the other using a high-speed planetary mixer. The prepared composite ink was tested for its flow behavior to understand the viscosity effect with the addition of coal. As seen in Figure S4, the viscosity and stress variations as a function of coal concentration did not display a uniform trend. Coal is a nonwettable and noncompressible solid that does not deform when subjected to shear force. This leads to the coal particles sliding against each other, which causes unpredictable behavior. The shear-thinning of the ink was conserved, regardless of the coal concentration.

The amplitude sweep results, depicted in Figure S5, revealed no significant variations in the linear viscoelastic regime, which is typically indicated by a sharp decline in both storage and loss moduli. In all instances, a strain value of approximately 0.5% marked the threshold for conducting frequency sweep measurements, essential for assessing the linear viscoelastic properties of the ink formulations. Figure S6 presents the frequency sweep outcomes for formulations with various coal concentrations. Similar to the flow sweep findings, though not distinct, subtle variation emerged with the changing coal amounts, likely attributable to the intrinsic characteristics of this solid material. As seen from the zoomed-in images in Figure S6a_2_,b_2_, *G*′ increased with coal concentration, i.e., 1 wt % ≪ 2 wt % ≪ 3, 5, and 7 wt % ≪ 9 wt %. Notably, the composite ink exhibited a predominately solid-like behavior, as evidenced by the higher storage modulus (*G*′) compared to the loss modulus (*G*″), underscoring the impact of the coal particles.

Figure 1b_1_ presents a simplified schematic of the DIW 3D printing process utilizing our specially formulated composite inks and outlining the curing process of the PF resin. The ink, comprising a blend of the resin, pTSA catalyst, and a homogeneous mixture of CB and coal (as depicted in Figure S7), was loaded into a syringe affixed to a mechanical extrusion-based print head, shown in Figure 1b_2_. The print bed temperature was set to 95 °C, and the printing parameters were meticulously fine-tuned through a series of trial-and-error experiments, as illustrated in Figure 1b_3_. Upon being deposited onto the heated bed with a glass substrate, the ink undergoes a rapid curing process, detailed in Figure 1b_4_ and elaborated upon in Section 3.1. It is important to note that all samples for the various tests were printed using these optimized parameters, regardless of the coal concentration.

### 2.3 Mechanical Testing

#### 2.3.1 Tensile Testing

**Figure 2:**
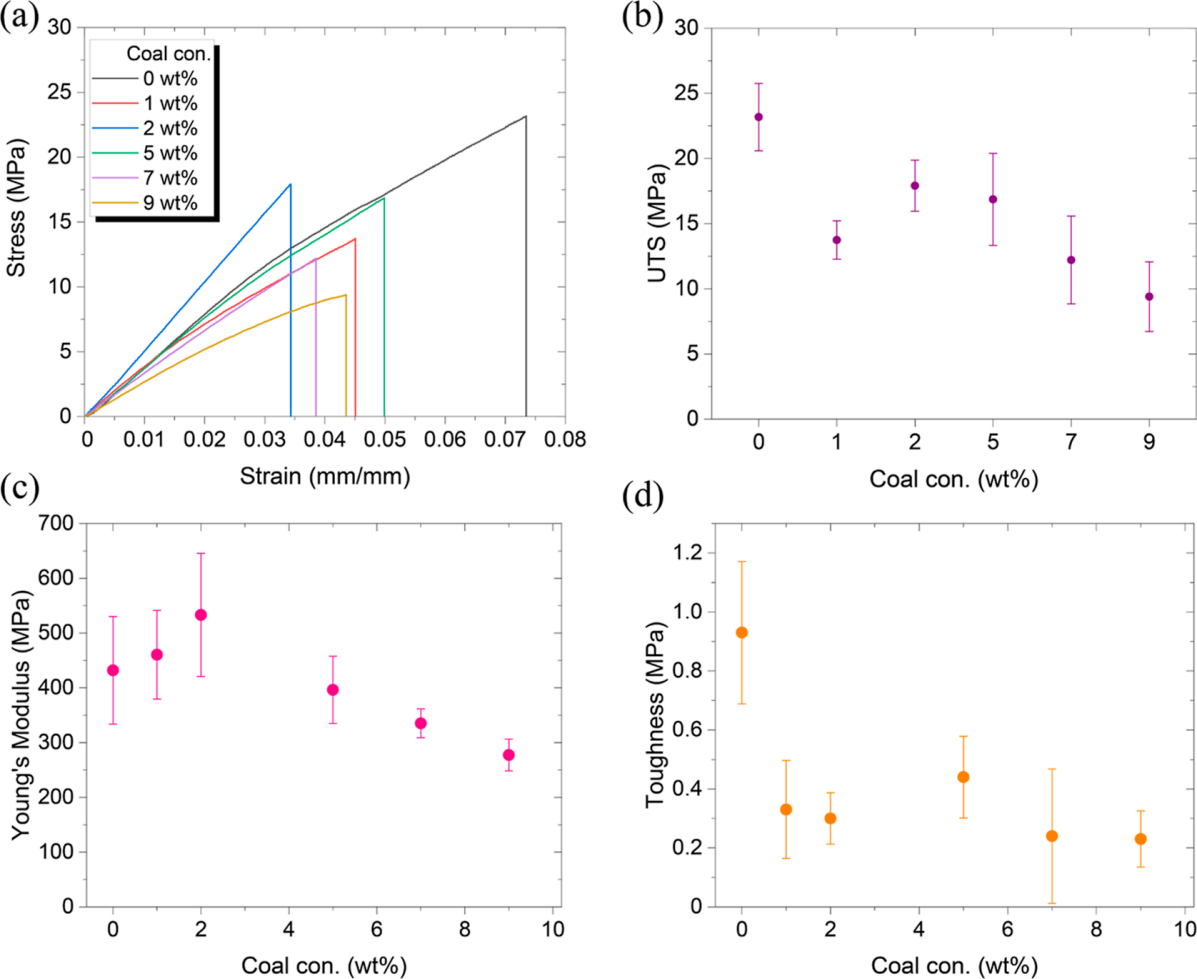
Tensile properties of the composites with different concentrations of coal: (a) displaying the stress–strain curves of the performed tests. Calculated values of (b) UTS, (c) Young’s modulus (*E*), and (d) toughness (*T*).

Figure 2a displays the stress–strain curves for samples tested with 2 wt % pTSA catalyst, 12 wt % CB, and various coal concentrations. It is noteworthy that, in comparison to the sample containing 0 wt % coal, all other samples exhibited a lower UTS, attributable to the inherent brittleness of coal. Interestingly, certain samples, particularly those with 1 and 2 wt % coal, demonstrated a higher modulus relative to the 0 wt % coal sample. The trend line suggests that the polymer of the cross-linked thermoset, irrespective of coal reinforcement, is likely to undergo brittle fracture. Additionally, the stress–strain data are instrumental in determining other mechanical properties such as toughness, typically represented by the area under the curve. The calculations for UTS (σ_max_) and Young’s modulus (*E*) were conducted using specific equations
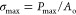
1
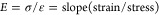
2where *P*_max_ is the maximum load achieved, *A*_o_ is the cross-sectional area, σ is the measured stress, and ε is the measured strain.

Figure 2a and Table S1 reveal that the UTS of coal–polymer composites is generally lower compared to the composite without coal. Among these composites, the sample containing 2 wt % coal displayed the highest UTS value. A similar trend was observed in terms of modulus (Figure 2b), where the 2 wt % coal sample surpassed all others in performance, including the composite without coal. A cursory examination of Figure 2a shows that a more brittle material, indicated by a steeper stress–strain curve slope, typically has a smaller area under the curve compared to a material with a lower modulus but higher strains. The toughness of the composites did not follow a distinct pattern as it is influenced by both the UTS and the modulus (*E*) (Figure 2c).

#### 2.3.2 Flexural Test

Similarly, flexural tests was performed for all the samples to quantify the flexural and bending properties. The flexural toughness is the area under the flexural stress–strain curve. The flexural modulus and bending strength were calculated based on eqs 3 and 4.
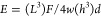
3
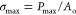
4where *L* is the span length, *F* is the load applied, *w* is the width of the sample, *h* is the height of the sample, *d* is the deflection of the sample, *P*_max_ is the maximum load achieved, *A*_o_ is the cross-sectional area, and ε is the measured strain.

**Figure 3:**
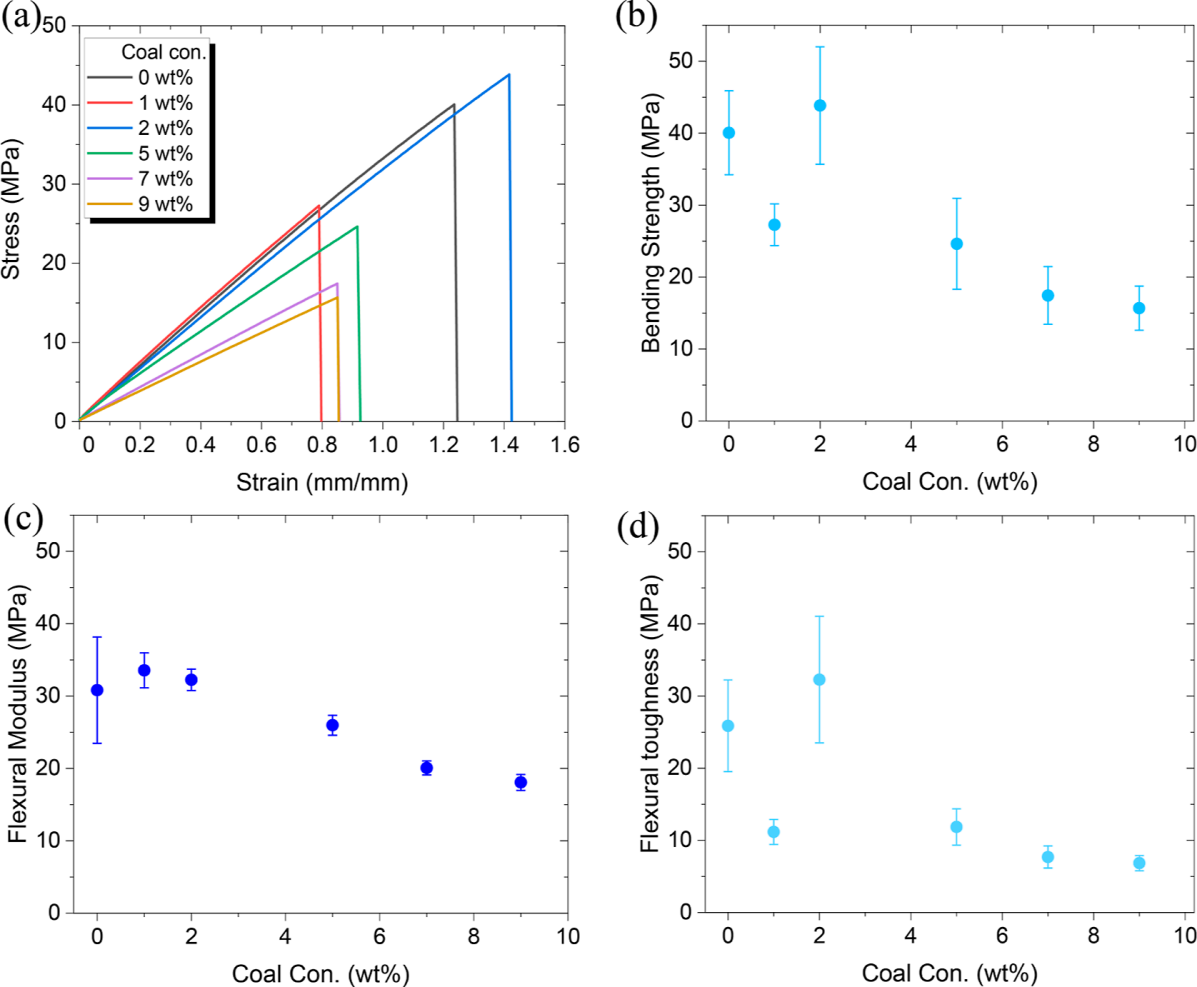
Flexural properties of the composites with different concentrations of coal: (a) displaying the flexural stress–strain curves of the performed test. Calculated values of (b) flexural ultimate strength, (c) flexural modulus, and (d) flexural toughness at different coal wt %.

As seen in Figure 3, which shows the flexural stress–strain, the 2 wt % coal composite outperforms all the other composites in strength, modulus, and toughness. Again, no significant differences in toughness were observed between composite samples with varying coal concentrations. Table S1 represents the quantitative values of the flexural test for all the tested samples.

#### 2.3.3 Effect of Coal Concentrations on the Mechanical Reinforcement

To comprehend the observed trends in tensile and flexural tests, it is essential to grasp the properties of the reinforcements integrated into the matrix. Coal, serving as a ceramic-type reinforcement, exhibits an anisotropic nature, implying a lack of preferential direction in properties and randomness in all directions. Notably, coal demonstrates high compression strength and a favorable modulus, albeit weaker tensile properties.^43,44^ According to the rule of mixtures, incorporating a reinforcement with a higher modulus into a matrix leads to an increase in the composite’s net modulus, aligning with the observed trend in the experimental results.^45^

As anticipated, the UTS of the composite declined with an increase in reinforcement concentration, consistent with the expected outcome given the weak tensile properties of the reinforcement. In contrast, concerning flexural properties, an augmentation in both modulus and flexural strength was noted. This phenomenon is attributed to the flexural loading scenario, where one-half of the sample, from the neutral axis, undergoes tension, while the other half experiences compression.^45^ The carbon reinforcement’s enhancement of the composite’s compression properties increased in flexural strength.

Higher concentrations of reinforcements lowered the mechanical properties of the composite throughout the board. As illustrated in Figure S8, overloading the matrix with higher concentrations of reinforcement leads to its agglomeration and reduces the distance between adjacent coal particles in the matrix, causing a buildup of unnecessary stress concentration and premature failure. The incorporation of coal particles into the polymer matrix makes the nanocomposites more brittle, as evidenced by the reduction in overall strength. This brittleness arises because the coal particles can act as stress concentrators within the matrix, facilitating crack initiation. However, these same particles also serve to impede the propagation of cracks once they form. This crack-arrest mechanism is due to the coal particles creating a tortuous path for crack propagation, requiring more energy for a crack to progress and thus leading to an increased strain of failure.

This can also be seen in the material distribution as shown in Figure S7. For example, in ink with 9 wt % coal, the distance between two coal particles is small enough so that the force passing through the interfacial gap will overload the stress significantly, leading to multiple failure points. The stress does not have an even path to pass through, which leads to stress concentration, contributing to failure. This phenomenon is observed in inks with coal concentration above 2 wt %. The interfacial distance becomes smaller and smaller giving rise to higher stress concentrations and ultimately premature failure. From these observations, it can be concluded that for coal with an average particle size of 250 μm, the reinforcement of 2 wt % coal displays the best results. It must be noted that based on our principle, the coal concentration may vary based on the particle size of reinforcements. For bigger coal sizes, the loading concentration must decrease and for lower coal sizes, the loading concentration can increase.

**Figure 4:**
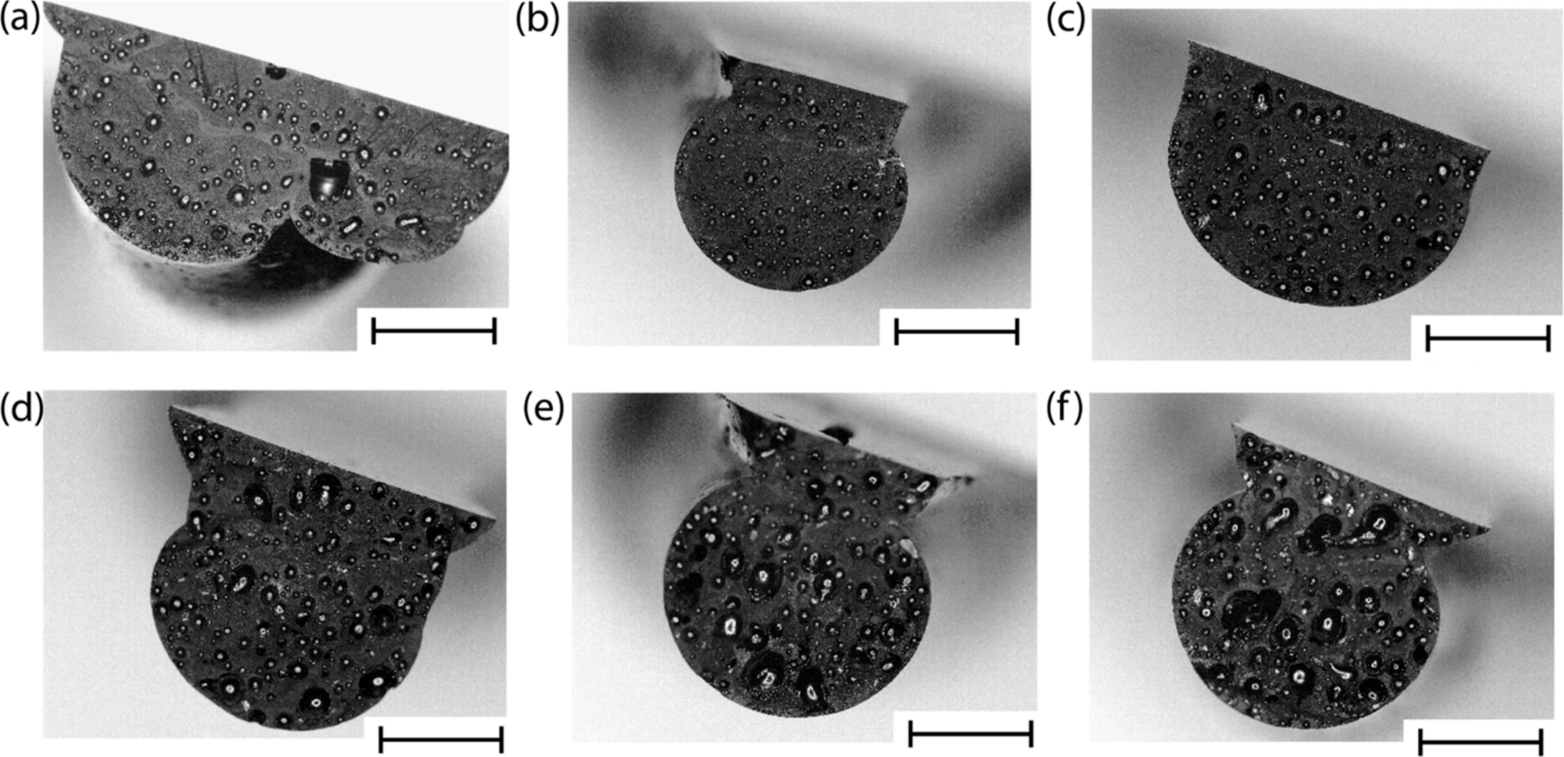
Coal-composite cross-section areas’ images by an optical microscope at different concentrations of coal (a–f) 0–9 wt % (scale bar 2 mm).

Pore size and porosity are indicators of 3D printing quality control. During the mechanical tests, voids in the composite were noticed. As visually seen in Figure 4, at low concentrations (0, 1, and 2 wt % coal) the size of the voids is small, and the distribution of the voids is even. However, at higher concentrations, the voids become significantly larger, and the distribution is uneven. Image analysis was conducted using optical images and ImageJ to calculate the pore area of all the samples. The details of the analysis are outlined in Table S2. Voids are one of the demerits of ink-based printing. It would be difficult to avoid them even without any reinforcements added.^46^ Also, the used polymer (J2027L phenolic resin) has about 10–15 wt % water content. The water is deemed to evaporate out of the resin while curing and will contribute to a small percentage of voids. But at higher concentrations of coal, some air is trapped between the coal particles, which will be included in the resin while mixing.

### 2.4 Structural Stability and Shape Fidelity

DIW has several advantages over the other additive manufacturing methods, as discussed previously.^26^ However, it has its limitations, like stability, dimensional accuracy, shape retention, ink preparation, rheological requirements, etc. So, it is important to understand the limitations of the ink formulation used in this research. Some test prints with different geometries were done to gauge the ability of our ink to print complex shapes and their accuracy.

**Figure 5:**
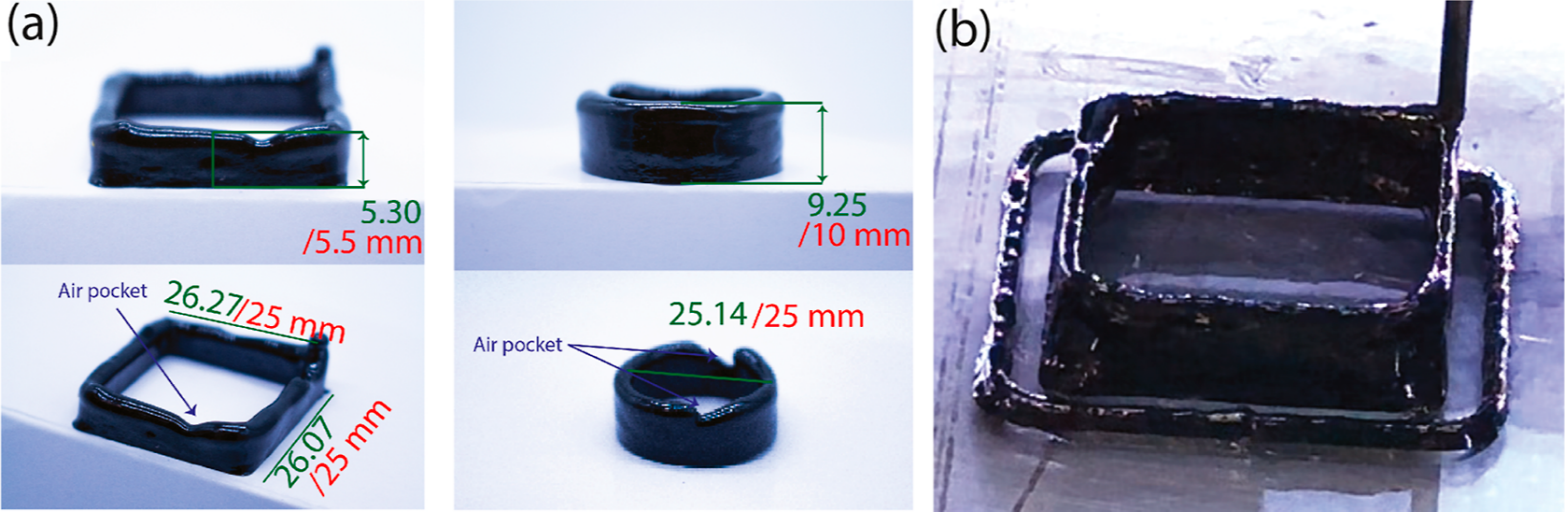
Printing accuracy of DIW: (a) coal composite; green color indicates the measured dimensions while red is the design dimensions. (b) Coal and MWCNT composite. MWCNT was added to increase viscosity.

As shown in Figure 5a, two thin wall structures were printed to assess the printability of free-standing structures. The shape of the print was accurate with a small deviation in the dimensions, namely, ∼1 mm in the *X* and *Y* directions and ∼0.5 mm in the *Z* direction. The main reason for these deviations was the limited heat propagation, i.e., thermal flux from the print bed in the *Z*-direction. Since the ink is thermally cured at 95 °C, the first few layers cure instantaneously. However, with an increasing height of the structures, the temperature propagation decreases, and the curing time increases. This leads to the sagging of the structure. One way to avoid this phenomenon is to ensure that the ink formulation possesses structural integrity through its surface tension or viscosity. This way the structure can self-support until the completion of the curing process.^30^ For example, adding high-viscosity additives like multiwalled carbon nanotubes (MWCNTs) can increase the maximum printable height considerably, as seen in Figure 5b.

## 3 Experimental Section

### 3.1 Materials

Cellobond J2027L (phenolic resole in aqueous solution, water content: 10–15%, free formaldehyde content: ≪1%, free phenol content: 6.5–10.5%) was purchased from Bakelite Synthetics, USA. The resin is temperature-reactive and thermally curable. Hence, it is stored in the refrigerator at ≪5 °C to extend its shelf life. The resin is kept at room temperature (RT) (approximately 22 °C) for approximately 30 min to attain its original viscosity before use. Anthracite coal was obtained from Naughty Boys Coal Company, USA. CB was obtained from Atlantic Equipment Engineers, Inc., USA. *p*-Toluenesulfonic acid monohydrate (pTSA) (ACS reagent, ≥98.5% and a molecular weight of 190.22 g/mol) was purchased from Millipore Sigma, USA, to be used as a curing catalyst. Luer lock assortment needles were obtained from Dispense All, USA. All materials and polymers were used as received.

### 3.2 Processing and Characterization

The resin and the reinforcements, viscosity modifier, and catalyst were added one after the other in order and mixed uniformly using a dual asymmetric planetary mixer (FlackTek DAC 330–100 L) from 1500 to 2500 rpm at a 500 rpm increment every 10 s. Curing analysis and kinetics studies of the resin and its mixtures were conducted using DSC (Discovery DSC 250, TA Instruments). The tests were performed using a hermetic pan and lid with a sample mass of 5–10 mg from 0 to 160 °C with a heating rate of 10 °C/min in an inert atmosphere (nitrogen).

Various concentrations of the acid catalyst (pTSA) were used to determine the optimal catalyst concentration for instantaneous curing/solidification of ink during 3D printing. The rheological studies were performed with a rheometer (Discover Hybrid Rheometer HR2, TA Instruments). All rheological tests were done at RT and in an air atmosphere using a disposable 25 mm aluminum parallel plate setup, with a 300 μm trim gap. A logarithmic flow sweep test was conducted with an increasing shear rate from 10^–3^ to 8000 s^–1^. The amplitude sweep was conducted from 10^–3^ to 1000% oscillation strain at a constant frequency of 1 Hz to obtain the limit of the linear viscoelasticity regime. The frequency sweep was conducted from 0.01 to 100 Hz at a constant strain of 0.5%.

TGA was conducted to evaluate the purity of coal from RT to 900 °C with a sample mass of 10–15 mg at a ramp rate of 10 °C/min in an inert atmosphere (nitrogen).

The dispersion quality of the reinforcement powders was examined using an optical microscope (Olympus MX50) and the cross-sectional images of the printed samples were captured with a 3D measuring microscope (Keyence V-3200).

Tensile tests were conducted using an Instron 3367 universal testing machine with a 2 kN load cell at a constant strain rate of 0.1 s^–1^. The samples for tensile tests were printed according to the American Society of Testing and Materials (ASTM) D638 Type V standard. The total length of the sample was 63.5 mm, the neck length was 9.53 mm, and the thickness was 3 mm. Results discussed in this study are an average of five samples for each test performed at RT.

The flexural tests were done using an ADMET’s eXpert 7600 uniaxial single-column testing machine with a 5 kN load cell and a constant strain rate of 1 min^–1^. The dimensions of the flexural samples were 50 mm in length, 5 mm in width, and 1.5 mm in height. Results discussed in this study are an average of five samples for each test performed at RT.

### 3.3 Printing Parameters and Process

Using the Hyrel Hydra 16A with an SDS10 (10 mL syringe module) print head, DIW 3D printing was carried out. Renowned for its versatility and modularity, the Hydra 16A provides a diverse array of attachments, ensuring optimal printability. The printing platform features a heated bed reaching temperatures of up to 120 °C, with a substantial print area measuring 60 cm × 40 cm × 25 cm in the *XYZ* planes, respectively. Several interconnected parameters, including print speed, extrusion rate, layer height, resin viscosity, and nozzle diameter, play crucial roles in the printing process. The machine’s precise quantitative control over the extrusion resin is not possible as it is automatically determined by the software. The software that controls the machine also calculates the volume of resin to be extruded per second based on parameters such as the printing speed, layer height, and nozzle diameter. The ink, loaded into the syringe with a 14-gauge nozzle (inner diameter of 1.32 mm), demonstrated optimal results at a printing speed of 7 mm s^–1^ and a layer height of 0.3 mm after thorough testing. Under these chosen parameters, the ink dispensing rate was 9.58 mm^3^ s^–1^, and the software automatically adjusted any variations in print speed or layer height. Following printing, the samples underwent curing and temperature soaking, utilizing a Thermo Scientific Lindberg/Blue M oven set at 90 °C for approximately 12 h, followed by 120 °C for about 8 h.

## 4 Conclusions

In summary, this study investigated the viability of employing coal as an economical, alternative, and sustainable reinforcement in ink-based polymer additive manufacturing. CB served as a viscosity modifier, aiding the dispersion of the denser coal particles. Notably, the inclusion of coal retained the desired shear-thinning rheological properties of the ink, preserving the crucial fluidic ink behavior necessary for DIW-based 3D printing. Planetary mixing ensured a uniform dispersion of particles in the ink, mitigating common issues like nozzle clogging. Despite noticeable pore formation in postcured samples, mechanical tests yielded promising outcomes at a 2 wt % coal concentration, indicating a 35% increase in tensile modulus, a 12.5% rise in the flexural strength, and a 5.4% increase in the flexural modulus compared to ink with no coal. Additionally, the successful 3D printing of thin-walled structures with simple geometric shapes, such as squares and circles, exhibiting minimal deviation from intended dimensions, indicates potential for manufacturing more intricate shapes in future works.

## Supporting Information Available

Graphical representations of DSC, rheological, TGA analysis, tensile and flexural stress–strain curves; tabulation of measured mechanical properties; ImageJ pore size analysis; optical images of coal distribution in the ink; and illustrations for discussing the effect of coal reinforcements (PDF)
