# Supplementary material for: Ink-Based Additive Manufacturing of a Polymer/Coal Composite: A Non-Traditional Reinforcement
Source: ACS Appl Eng Mater. 2024 May 6;2(5):1315–23. doi: 10.1021/acsaenm.4c00126 (PMC11129187; doi:10.1021/acsaenm.4c00126)
Supplement: Supplementary file 1 — em4c00126_si_001.pdf [file em4c00126_si_001.pdf]

Supporting Information

Ink-Based Additive Manufacturing of Polymer/Coal Composite: A Non-Traditional Reinforcement

Barath Sundaravadivelan,[†,‖] Dharneedar Ravichandran,[‡,‖] Anna Dmochowska,[¶] Dhanush Patil,[‡] Sri Vaishnavi Thummalapalli,[§] Arunachalam Ramanathan,[§] Jorge Peixinho,[¶] Guillaume Miquelard-Garnier,[¶] and Kenan Song[*,‡,§]

†*Department of Mechanical Engineering, School for Engineering of Matter, Transport, and Energy, Ira A. Fulton Schools of Engineering, Arizona State University, Tempe, AZ, USA 85281*

‡*School of Manufacturing Systems and Networks, Ira A. Fulton Schools of Engineering, Arizona State University, Mesa, AZ, USA 85212*

¶*Laboratoire PIMM, CNRS, Arts et Métiers Institute of Technology, Cnam, HESAM Universite, 75013 Paris, France*

§*School of Environmental, Civil, Agricultural, and Mechanical Engineering (ECAM), College of Engineering, University of Georgia, Athens, Georgia, USA 30602*

‖*Co-firstauthor*

E-mail: kenan.song@uga.edu

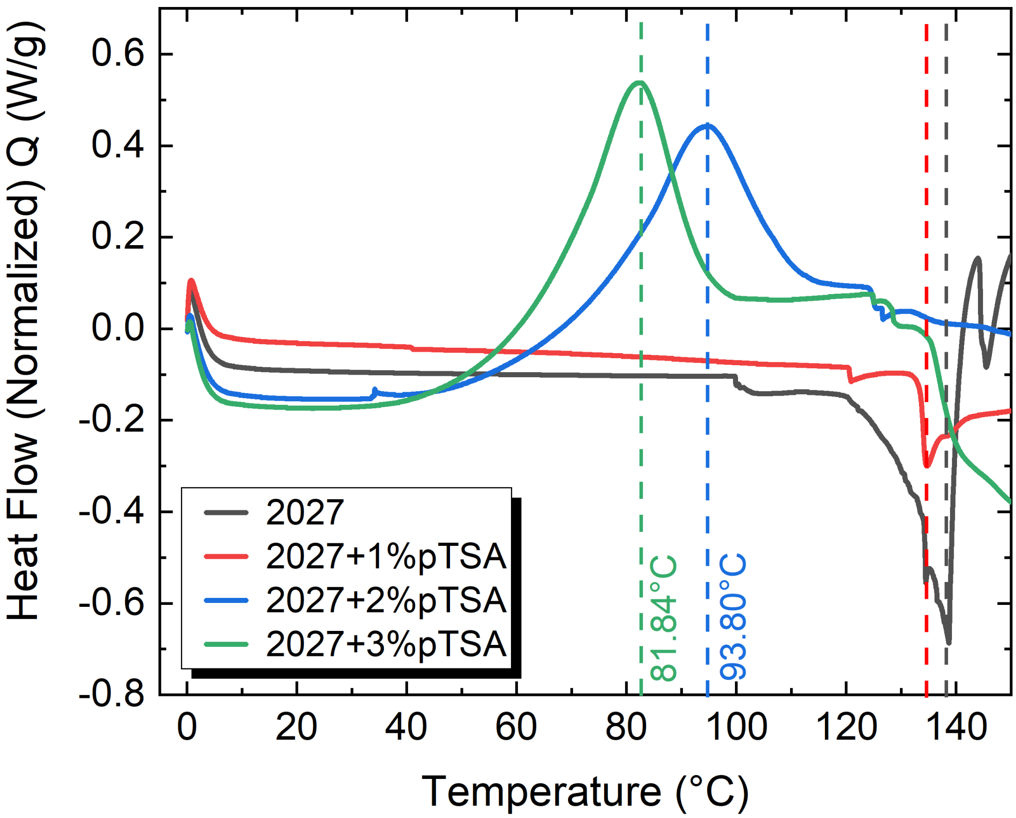

Figure S1: DSC curability studies of the resin without and with various concentrations of pTSA.

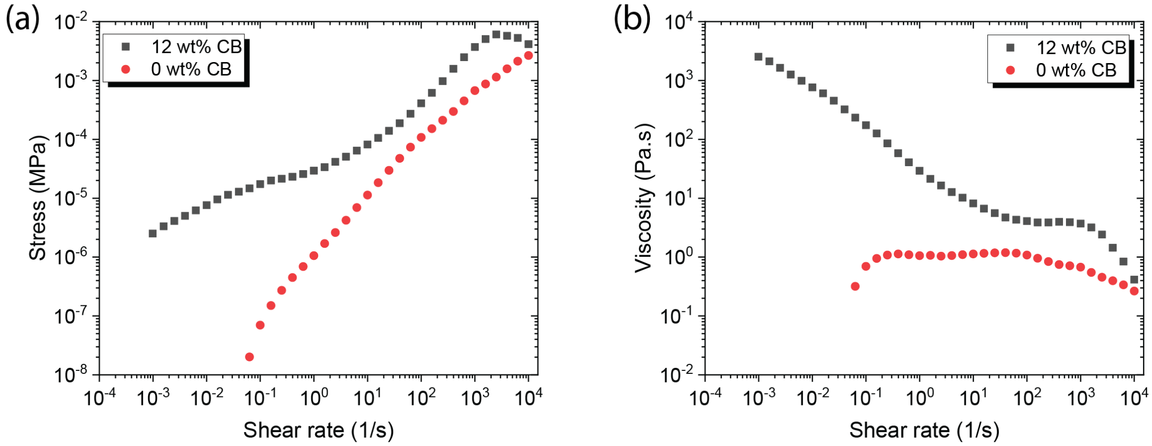

Figure S2: Rheology analysis of resins containing different additive fillers (i.e., 12wt% vs. 0wt% CB) to analyze the flowability of the resin and optimize the minimum concentration of the viscosity modifier (CB) required during printing, (a) shear rate vs. stress and (b) shear rate vs viscosity.

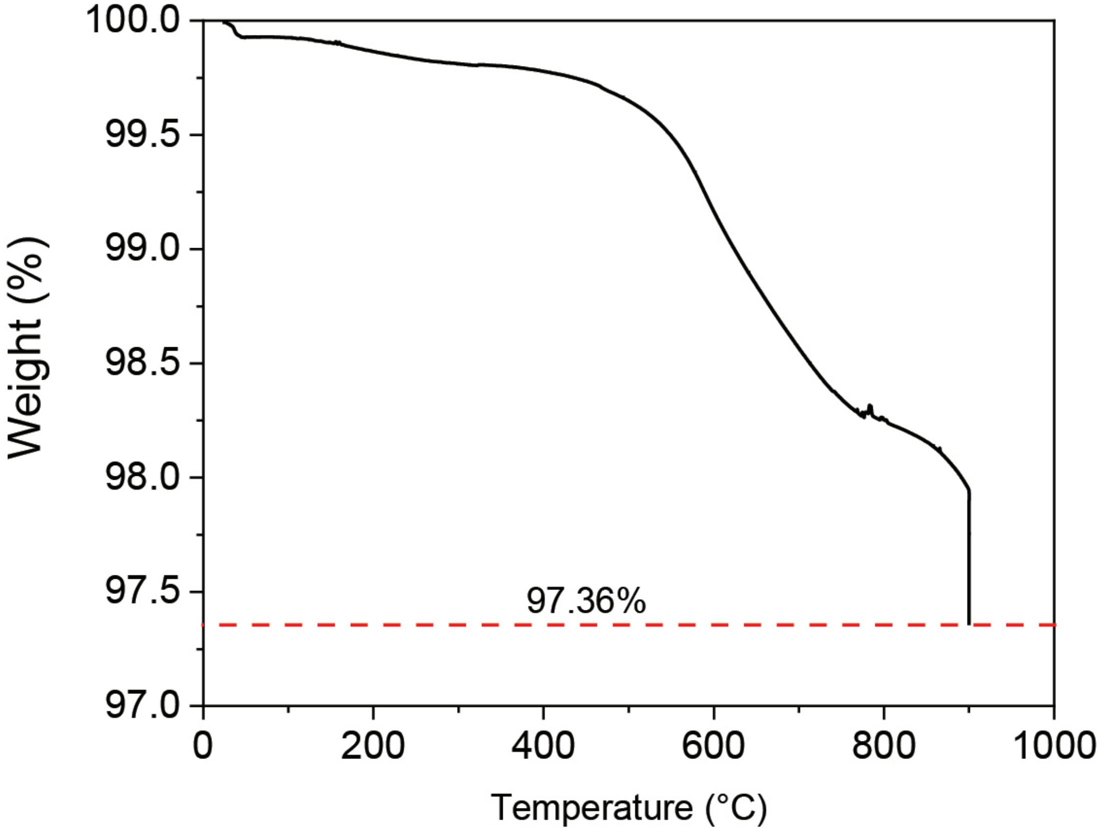

Figure S3: TGA analysis showing the purity of coal used in this study

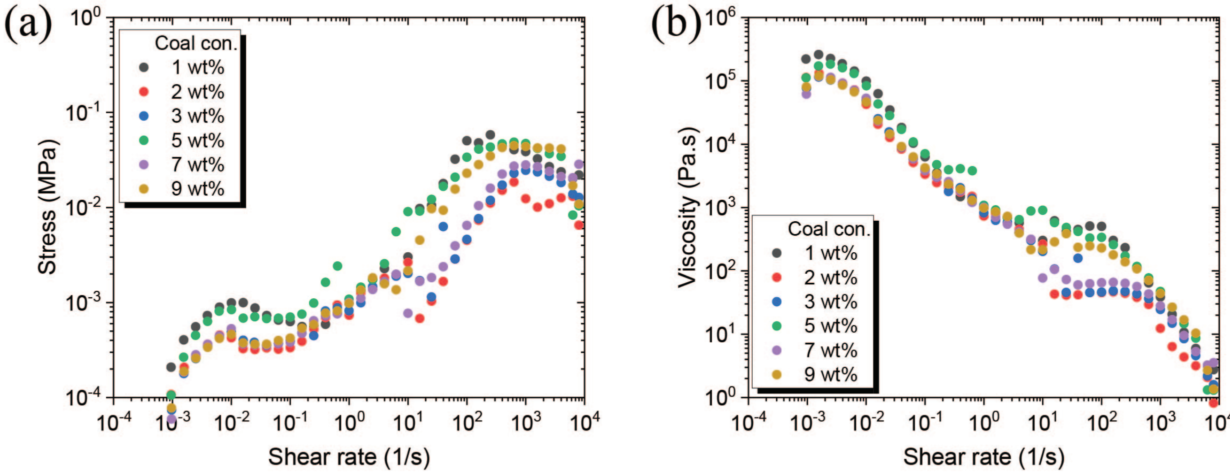

Figure S4: Flow sweep of resins with different concentrations of the coal with the (a) shear stress vs. shear rate and the (b) viscosity vs shear rate.

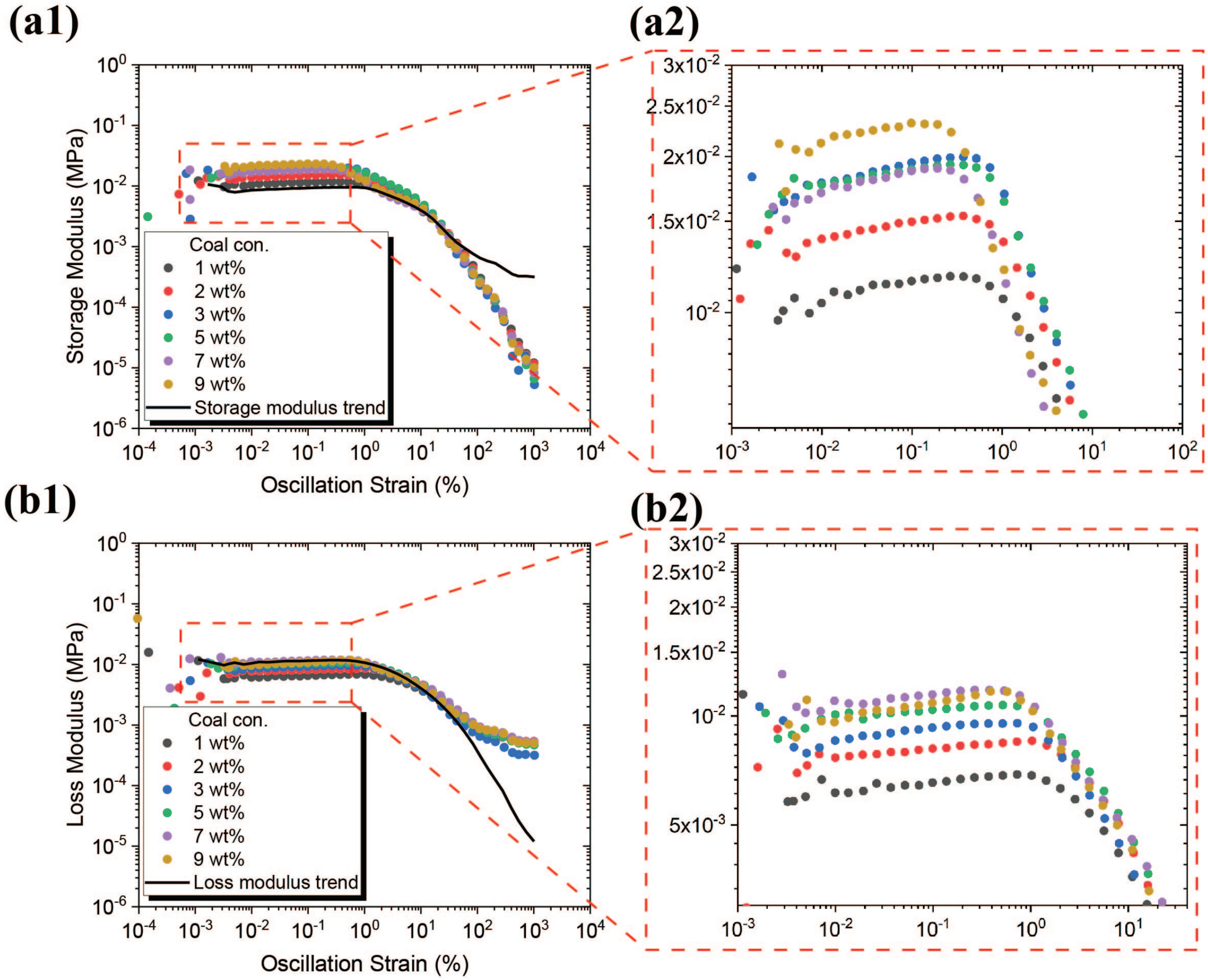

Figure S5: Amplitude sweeps of the resin with different concentrations of coal. (a1) Storage modulus vs oscillation strain, (a2) storage modulus trend at low oscillation strain, (b1) loss modulus vs oscillation strain, and (b2) loss modulus trend at low oscillation strain.

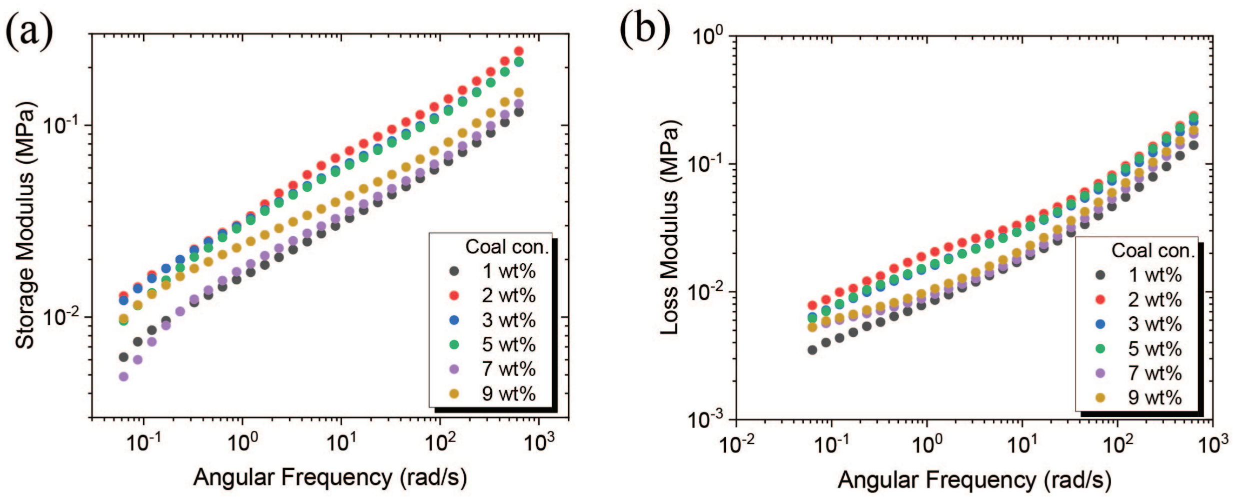

Figure S6: Frequency sweep of the resin containing different concentrations of coal with the (a) storage modulus vs angular frequency and (b) loss modulus vs angular frequency.

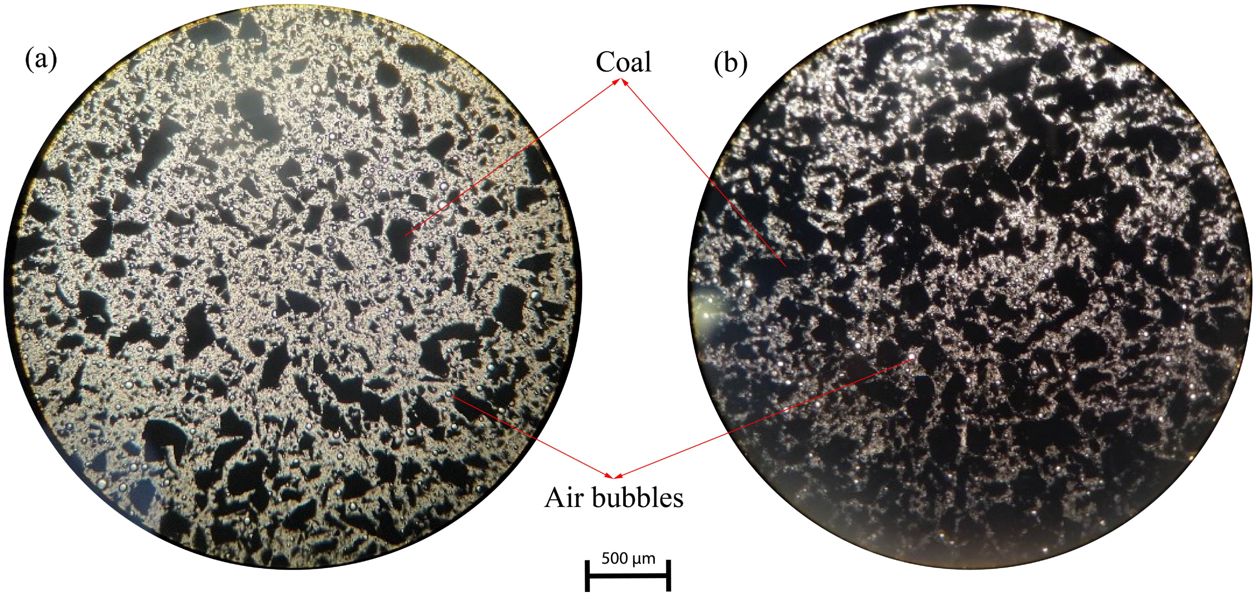

Figure S7: (a) 2 wt% and (b) 9 wt% coal distribution in the resin.

Table S1: Tabulation of average values of mechanical properties of tensile and flexural testing

**Coal con.**	**Tensile Test**			**Flexural Test**		
(%)	UTS (MPa)	Modulus (MPa)	Toughness (MPa)	Bending strength (MPa)	Modulus (MPa)	Toughness (MPa)
0	23.18	431.97	0.93	40.06	30.81	25.87
1	13.75	460.50	0.33	27.27	33.55	11.17
2	17.91	533.11	0.30	43.85	32.24	32.28
5	16.86	396.29	0.44	24.63	25.97	11.85
7	12.21	335.16	0.24	17.44	20.07	7.69
9	9.40	277.36	0.23	16.58	18.07	6.84

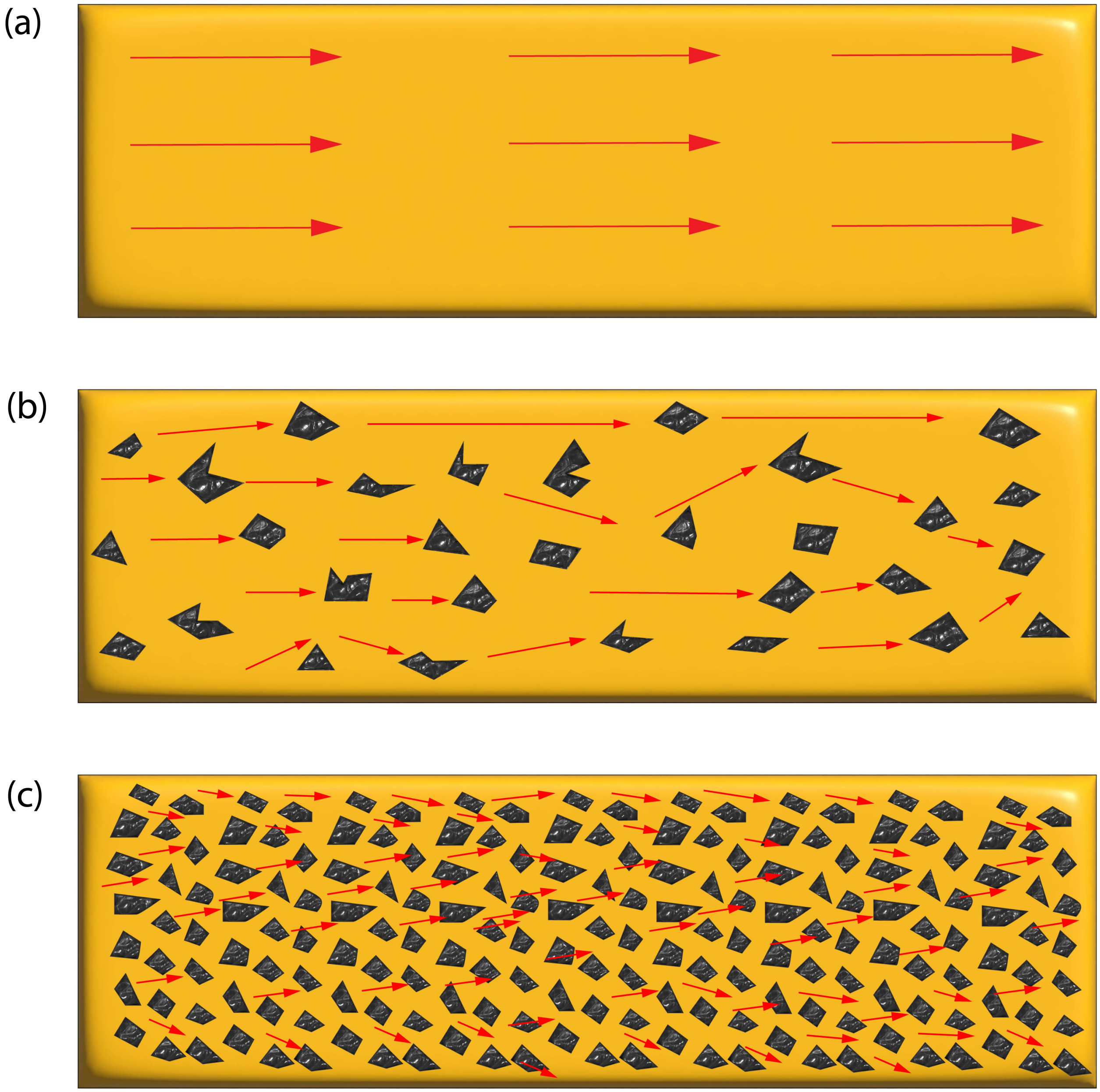

Figure S8: Stress distribution (represented by the red arrows) for samples made of (a) pure resin (b) with a low concentration of coal, and (c) with a high concentration of coal.

Table S2: The pore area of individual samples analyzed using ImageJ.

**Coal conc. (%)**	**Min. pore area (mm^2)**	**Max. pore area (mm^2)**	Avg. pore area (mm^2)
0	1.37E-5	0.20	1.30E-4
1	1.37E-5	1.35	1.95E-4
2	1.37E-5	4.03	1.73E-4
5	1.37E-5	4.50	4.82E-4
7	1.37E-5	4.20	5.86E-4
9	1.37E-5	5.87	6.32E-4

The analysis was conducted following the protocols outlined on the official ImageJ website. The pore sizes presented in Table R1 are based on the scale of the optical images. Across all samples, the minimum measured pore area was 1.37E-5 $\mathbf{mm^2}$ also due to the characterization limits. As anticipated, an increase in coal concentration resulted in a rise in average pore area, with the 2 wt% coal sample exhibiting the lowest among all concentrations.

It is crucial to note that the pore sizes determined using the software solely account for surface porosity and may not always reflect precise measurements. To address this limitation, we attempted BET porosity analysis. However, the accuracy of this analysis is contingent upon the sample's gas absorption capacity, especially considering the lack of capability to detect pores above the microscale. Unfortunately, the samples subjected to BET analysis did not yield any discernible results.
